# Correction

**DOI:** 10.1080/10495398.2026.2660452

**Published:** 2026-04-23

**Article title:** Mapping the proteome landscape of Indian Zebu (Sahiwal) spermatozoa using high-resolution mass spectrometry and in-silico annotation

**Authors:** Bhusan, V., Ali, S. A., Parasar, A., Kumar, S and Mohanty, A. K

**Journal:** *Animal Biotechnology*

**Bibliometrics:** 35(1):2428402

**DOI:** https://doi.org/10.1080/10495398.2024.2428402

When the article was published online, Figure 1 was included to provide qualitative microscopic confirmation of sperm health and integrity prior to proteomic analysis, illustrating staining patterns indicative of viability, non-capacitated status, and non-acrosome-reacted spermatozoa. The figure served as a supportive visual reference and does not constitute experimental or quantitative data used in the proteomics analyses reported in the study.

It has since been identified that the images depict buffalo spermatozoa and were therefore incorrectly labeled as Sahiwal (*Bos indicus*) cattle spermatozoa in the published article. This figure did not contribute to or affect the proteomics workflow, mass spectrometry data, bioinformatic analyses, interpretations, or conclusions reported in the study.

Figure 1. Has been removed from the article.

